# Spectral and Texture Properties of Hydrophobic Aerogel Powders Obtained from Room Temperature Drying

**DOI:** 10.3390/molecules26061796

**Published:** 2021-03-23

**Authors:** Dimitar Shandurkov, Petar Ignatov, Ivanka Spassova, Stoyan Gutzov

**Affiliations:** 1 Faculty of Chemistry and Pharmacy, Department of Physical Chemistry, University of Sofia St. Kliment Ohridski, 1164 Sofia, Bulgaria; dimitar.shandurkov@gmail.com (D.S.); pepi.ignatov@gmail.com (P.I.); 2 Bulgarian Academy of Sciences, Institute of General and Inorganic Chemistry, 1113 Sofia, Bulgaria; ispasova@svr.igic.bas.bg

**Keywords:** sol-gel, aerogel, IR, adsorption, fractal dimension

## Abstract

Attenuated Total Reflectance Infrared (ATR-IR) spectroscopy and texture measurements based on nitrogen adsorption-desorption isotherms are combined to characterize silica aerogel granules with different degrees of hydrophobicity. The aerogels were prepared from tetraethoxysilane via a room temperature hydrolysis-gelation process, solvent exchange, hydrophobization, and drying at subcritical conditions. The dependencies between the texture properties, pore architectures, surface fractal dimensions, and degree of hydrophobicity of the samples are extracted from the ATR-IR spectra and the adsorption-desorption isotherms. The IR absorption in the region of the Si-O-Si and Si-OH vibrations is used for a description of the structural and chemical changes in aerogel powders connected with their surface hydrophobization. The Frenkel–Halsey–Hill (FHH) theory is applied to determine the surface fractal dimension of the powder species.

## 1. Introduction

The sol-gel process is a versatile approach to manufacturing a wide variety of materials and tailoring their properties [1]. The technique is used to produce metal oxide gels, highly porous materials, optical materials, thin films, coatings, hybrid organic-inorganic materials, drug delivery systems, optical filters, etc. [2]. From a physico-chemical point of view, the sol-gel process allows for the homogenization of the precursors at a molecular level and the subsequent easy control of the rate of hydrolysis and condensation (gelation) reactions by varying the catalyzing agents, sol volume, temperature, and drying conditions. A special class of sol-gel materials is aerogels, which are known for their extremely low densities (ranging from 0.001 to 0.5 g/cm^3^), low optical refraction index (1.002), low thermal conductivity (0.02 W/m·K), speed of sound through a material (70 m/s), and a relative dielectric constant 1.008 at 3–40 GHz [3].

In addition to the classical aerogel-based thermal and sound insulation materials, optical materials with sensory [4] and biological applications [5,6] constitute a new, fast-growing field of aerogel development. In a recent paper, the possibilities for the incorporation of optically active hybrid molecules in hydrophobic aerogel granules were discussed [7]. In the same paper, a useful approach for the impregnation of hydrophobic aerogel granules with [Tb(phen)_2_](NO_3_)_3_ complexes was described using high-porosity aerogel granules and powders, at about 95%. Colloidal techniques for the preparation of impregnated porous silica for optical purposes were discussed in [8].

There are two general strategies for the preparation of silica aerogel granules and powders: supercritical drying and subcritical drying [1,2]. Moreover, the properties of silica aerogels can be additionally improved using freeze-drying conditions [9]. Recently, a novel approach based on 3D printing, leading to a high specific surface area and bulky aerogels, was demonstrated [10].

The subcritical synthesis of oxide aerogels from alcoxydes consists of few main steps: the hydrolysis of the alcoxide species in acidic conditions until oligomers form, alkaline polycondensations, gelation, and drying subcritical conditions. Choosing hydrocarbon-containing silica precursors for treating wet gel with surface-modifying agents such as trimethyl chlorosilane (TMCS) leads to the production of hydrophobic gels, granules, powders, and coatings with different degrees of hydrophobicity [7,11]. In this way, significant amounts of highly porous powders can be prepared depending on the drying capacity. The subcritical production procedure needs a strong development of physico-chemical characterization in order to improve the process preparation conditions and ensure the reproducibility of the prepared aerogel samples.

Although classical methods such as wetting and contact angle techniques, thermogravimetry, thermal conductivity, etc. [12,13,14,15], are used to study the surface and bulk properties of silica glasses, it has been shown that vibrational spectroscopy has significant potential in determining the structure of building species and even the stress (angle distribution of neighboring tetrahedra) and imperfections of the glassy network [11,16]. Infrared (IR) spectroscopy is a widely used method to characterize structure-forming species in silica materials. The first theoretical description of the vibrational spectra of AX_2_ tetrahedral glasses, which are SiO_2_ in part, was achieved by Sen and Thorpe [17] and leads the so-called Central Force model. Later, this model was refined and limited by Galeener [18]. The model assumes the glass building block is AX_4_ regular tetrahedral (all A-X bonds are the same length and X-A-X angles have the same value—around 109.5 degrees). The main variables are the mass of the cation (A), the mass of the anion (X), and the A-X-A inter tetrahedral angle, varying from 90 to 180 degrees. With these assumptions in mind, the model takes into account only the closest neighbor interactions and local order; however, it incorporates certain elements of disorder, since it is not periodic in space and the dihedral angles may take any value. However, this model does not describe all vibrational bands in some glasses and one should consider other types of interactions, such as long-range-acting Coulomb forces [19].

Despite the large number of investigations of hydrophobic aerogel powders with a potential application as insulation fillers, a lack of knowledge exists about combining IR spectroscopy and texture measurements to describe the microstructure of hydrophobic silica aerogel-like powders. The aim of this contribution is to combine texture and IR-spectroscopic measurements to find the relations between the pore architecture of the aerogels, texture properties, and spectral features, depending on the degree of hydrophobicity.

## 2. Results and Discussion

### 2.1. Physical Properties

It is evident that hydrophobization leads to a decrease in the bulk density of the aerogel materials here. A similar bulk density (0.12 g/cm^3^) for room temperature-dried samples and solvent exchange 48 h at room temperature was recently discussed [15,20].

Here, the degree of hydrophobicity α, is expressed as:

(1) $$\alpha =\frac{n_{TMCS}}{n_{TEOS}}.$$

### 2.2. ATR-IR Spectroscopy

**Figure 1:**
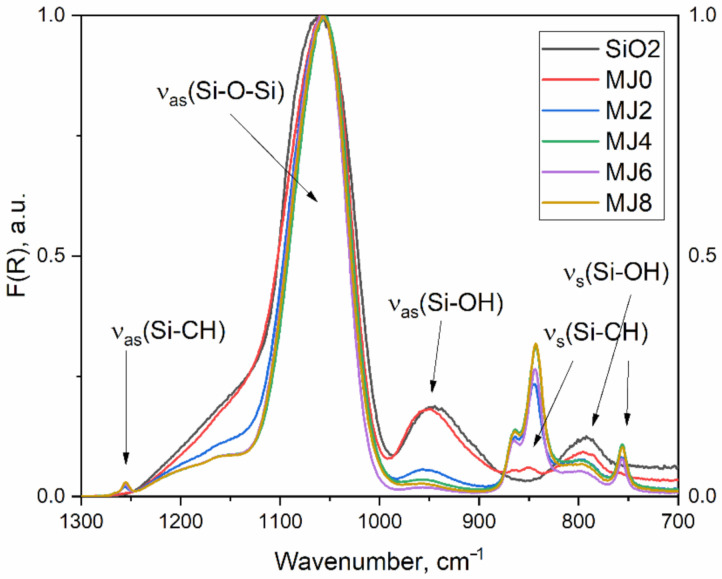
Normalized ATR-IR spectra of the studied samples. Sample notations are the same as in Table 1: MJ0—α = 0; MJ2—α = 0.352; MJ4—α = 0.700; MJ6—α = 1.055 and MJ8—α = 1.407. The SiO_2_ represents bulk silica.

All synthesized samples display the abovementioned bands characteristic of SiO_2_. The spectra of samples MJ0, MJ2, MJ4, MJ6, and MJ8 and reference sample SiO_2_ (dense amorphous silica) are shown in Figure 1. It is well known that the SiO_2_ IR spectrum has a few distinct bands. In the longer wavelength region, it displays a broad peak (3600–3000 cm^−1^) associated with the ν(O-H) stretching modes of hydroxyl groups, appointed to residual water, alcohols, or Si-OH groups. The presence of water or ethanol can be further confirmed or disproved by their IR active modes at 1630 and 1274 cm^−1^. Interest has been aroused in the so-called finger-print region in the vibrational spectrum of SiO_2_. This region, located in the 1300–700 cm^−1^ interval, provides information on the structural characteristics of the material. The region mentioned could be additionally divided into two sub regions—namely, the 1300–900 cm^−1^, which contains the ν_as_ (Si-O-Si) and ν_as_(Si-O-H) bands, and the 900–700 cm^−1^, where the ν_s_(Si-O-Si) and ν_s_(Si-CH_3_) vibrations also are located. The broad 1200–1000 cm^−1^ band is said to be comprised of four overlapping components: the transvers (TO) and longitudinal (LO) optic modes of the four- and six-member siloxane rings (SiO)_4_ and (SiO)_6_. The LO mode of the six- and four-membered rings LO_6_ and LO_4_ is centered around 1220 and 1150 cm^−1^, respectively. The transversal modes of the six- and four-membered rings LO_6_ and LO_4_ are, respectively, centered at 1050 and 1080 cm^−1^. The LO/TO splitting, which arises from the long-range Coulomb forces, for the four- and six-membered rings, is between 80 and 140 cm^−1^. The six-membered siloxane rings are less tensioned, with an average inter tetrahedral angle of 140°. The four-membered siloxane rings are more tensioned, with an average angle of 120°. However, they are thermodynamically more stable. The six-membered siloxane rings are usually linked to higher porosity of the material. The position of the LO mode is strongly affected by the environment and can shift more than the TO mode. Separating the four overlapping peaks and extracting information from the spectrum requires deconvolution by using non-linear least square fit of 4 Gaussian functions in this region [11,16,21,22,23,24]. Distinct peaks can be found around 1260, 860, and 760 cm^−1^, corresponding, respectively, to antisymmetric ν_as_ (Si-CH_3_) and symmetric ν_s_(Si-CH_3_) vibrations in surface-modified silica [25,26]. The 950 cm^−1^ is assigned to the ν_as_(Si-OH) stretching vibration, the 800 cm^−1^ band is ascribed to symmetric ν_s_(Si-O-Si) vibrations. A pure SiO_2_ sample, amorphous silica, is used as a reference. The spectrum of the hydrophilic sample here (MJ0) is very similar to that of the reference sample SiO_2_. All the samples display a broad band at 1250–1000 cm^−1^ with a shoulder at the bigger wavenumbers and a maximum around 1060 cm^−1^.

**Figure 2:**
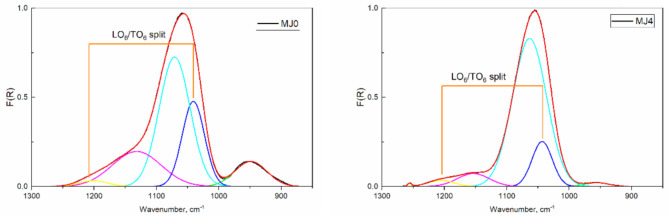
Spectral deconvolution of samples with a different hydrophobization degree: MJ0—hydrophilic silica, left; MJ4—α = 0.7, right. The appearance of the 1260 cm^−1^ band is indicative after the addition of the surface-modifying agent TMCS.

The spectral deconvolution is shown in Figure 2. For each band the area, the full width at half maximum (FWHM), position, and height are calculated; the respective data are given in Table 2.

The TO/LO splitting for the six-membered rings is closer to the theoretical 160 cm^−1^ splitting for α-quartz than the calculated 140 cm^−1^ for amorphous silica [16].

**Figure 3:**
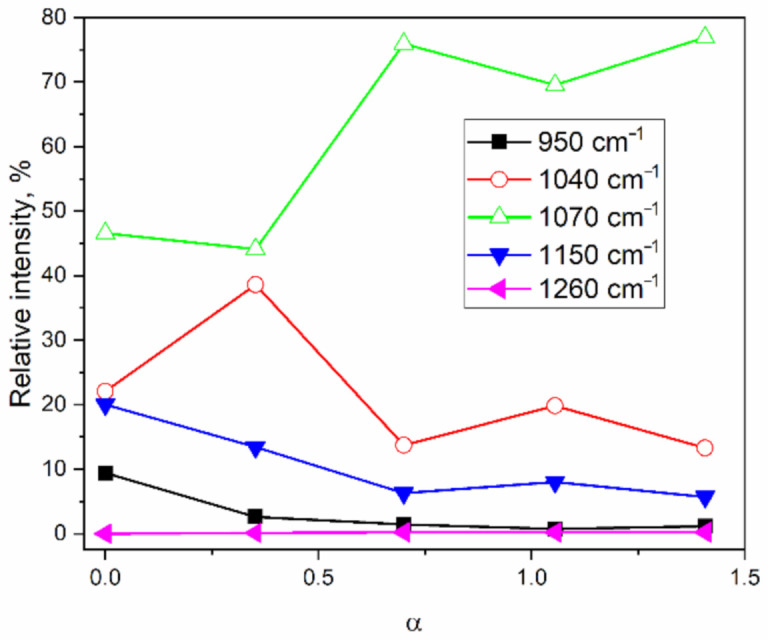
The relative area of the IR-bands band of samples in the region of the Si-O-Si and Si-OH vibrations.

It is visible (Figure 3) that the intensity of the 1150 cm^−1^ shoulder decreases with the increasing TMCS/tetraethoxysilane (TEOS) molar ratio, and can be explained by a rearrangement of four-membered rings during hydrophobization. A 1260 cm^−1^ peak is present for the surface-modified samples with TMCS (samples MJ2-MJ8, Table 1). The intensity of the 950 cm^−1^ peak, assigned to ν_as_(Si-OH), decreases with the addition of TMCS, which could be explained by the high reactivity of the surface-modifying agent towards –OH groups. The hydrophilic hydroxyl groups are partially replaced by –Si-(CH_3_)_3_ groups, which make the surface hydrophobic, giving rise to the 1260, 860, and 760 cm^−1^ bands (Figure 1) and decreasing the concentration of –OH groups, hence reducing the intensity of the 950 cm^−1^ band. The partial replacement of –OH groups by the bulky –Si-(CH_3_)_3_ groups is probably due to a steric hindrance.

### 2.3. Texture Properties

**Figure 4:**
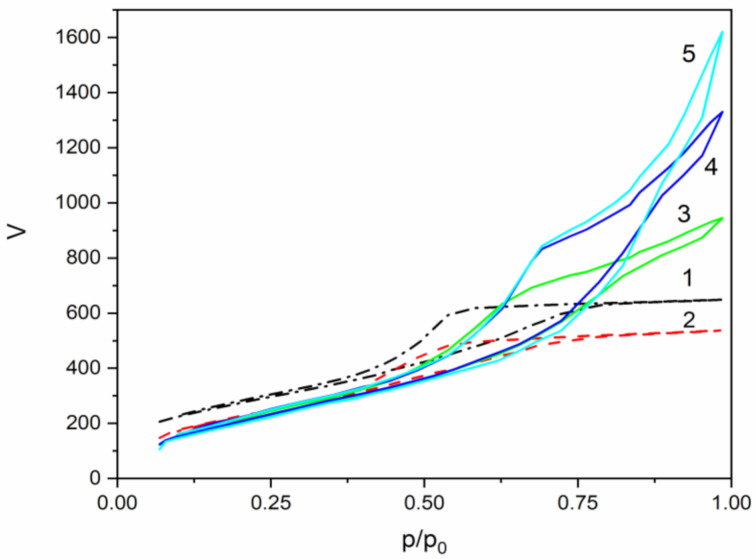
Low-temperature nitrogen adsorption-desorption isotherm of silica samples having a different degree of hydrophobicity: 1—MJ0, α = 0; 2—MJ2, α = 0.352; 3—MJ4, α = 0.700; 4—MJ6, α = 1.055 and 5—MJ8, α = 1.407.

Low-temperature nitrogen adsorption-desorption isotherms of samples MJ0, MJ2, MJ4, MJ6, and MJ8 were obtained at 77 K. The isotherms are shown in Figure 4. All the samples exhibit type IV isotherm, which, according to IUPAC classification, is associated with mesoporous materials (pore diameter distribution between 2 and 50 nm) [21].

Samples MJ0 and MJ2 display a low closure point of the hysteresis loop. Additionally, samples MJ0 and MJ2 exhibit an H2 hysteresis loop, which is common for amorphous glasses and inorganic gels; it used to be ascribed to bottleneck-shaped pores, but it is now recognized that this is a simplified model and network interactions should also be taken into account. Strongly hydrophobic samples MJ4, MJ6, and MJ8 do not display the characteristic limiting adsorption at high relative pressures. Their hysteresis loop resembles more the type H3 loop, which is associated with plate-like particles giving rise to slit-like pores [27,28,29,30]. In this way, the 77 K BET hysteresis loop leads to conclusions about the pore architecture depending on the TMCS/TEOS molar ratio.

**Figure 5:**
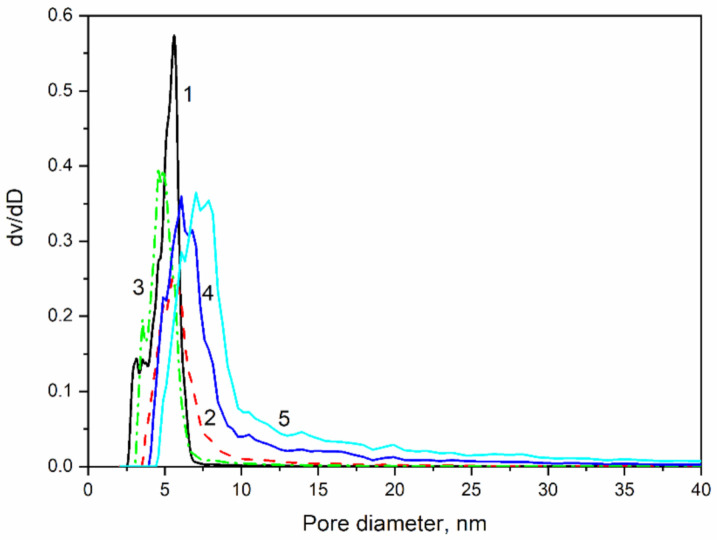
Pore size distribution of silica samples with different degrees of hydrophobicity: 1—MJ0, α = 0; 2—MJ2, α = 0.352; 3—MJ4, α = 0.7; 4—MJ6, α = 1.055 and 5—MJ8, α = 1.407.

The pore size distributions of the aerogel-like powders are shown in Figure 5. Samples MJ0, MJ2, and MJ4 show a narrow pore size distribution, but species MJ6 and MJ8 exhibit a wider pore size distribution with a shoulder at the bigger pore diameters. There is also a shift towards wider pore diameters with the increase in TMCS concentrations.

The texture properties of the samples are summarized in Table 3. A decrease in the specific surface (S_BET_) area with an increase in the TMCS/TEOS molar ratio is observed. Generally, the specific pore volume (V_t_) in the series increases together with the average pore diameter (D_av_). The bulk powder density decreases with the addition of TMCS (Table 1). Making the surface hydrophobic reduces the surface tension of the liquid inside the pores and reduces the stress induced by the evaporating fluid. Thus, fewer pores collapse during the drying process, leading to a less densified material. Therefore, more hydrophobic samples have wider pore size distributions, with a shoulder at the bigger diameters. Generally, smaller pores are linked to more developed surface, hence the bigger specific area of hydrophilic sample (MJ0). In samples MJ6 and MJ8, the smaller pores might be occupied by the bulky Si-(CH_3_)_3_ groups.

Many studies have shown that silica materials exhibit a fractal structure in a certain length scale. The number of molecules N_m_ of diameter d, required to fill a monolayer, is proportional to d^D_s_^, where D_s_ is the fractal surface dimension. For a flat surface, D_s_ is equal to 2 and for a space filling surface it approaches 3 [31,32,33,34]. The Frenkel–Halsey–Hill (FHH) theory describes the multilayer adsorption of a gas on a surface. The classical FHH equation for a flat surface reads:

(2) $$\frac{N}{N_{m}}=\frac{C}{kT}{\left[-\ln\left(\frac{p}{p_{0}}\right)\right]}^{-1/3},$$

where N is the number of adsorbed molecules, N_m_ is the number of molecules of certain diameter required to cover a monolayer, $\frac{p}{p_{0}}$ is the relative pressure, k and T are the Boltzmann constant and the absolute temperature, respectively, and C is a parameter describing the energy of adsorption and the adsorbate molecule volume. The fractal FHH isotherm adapted by Pfeifer et al. [34,35,36,37,38] takes the form:

(3) $$\frac{N}{N_{m}}=\frac{1}{3-D_{s}}\left[\frac{C}{kT}\ln{\left(\frac{p}{p_{0}}\right)}^{-1/m}-\left(D_{s}-2\right)\right],$$

and

(4) $$m=\frac{s}{3}-D_{s}.$$

The parameter s describes the isotherm shape and its theoretical value is equal to 3. The fractal FHH isotherm is reduced to the classical FHH isotherm for a flat surface (D_s_ = 2).

Neimark [39,40], Sahouli [41], and Venkatrman [42] argued that the value s = 2.24 gives satisfactory results for mesoporous inorganic oxides. The adsorbed quantity N/N_m_ could be substituted by the volume V of the adsorbed gas. A linear plot can be achieved by plotting lnV vs. $\ln\left[\ln\left[\frac{p}{p_{0}}\right]\right]$ and the fractal surface dimension D_s_ can be calculated from Equations (3) and (4). In this way, the surface fractal dimensions of the investigated samples is calculated (Table 2). The values of D_s_ are calculated by accounting for adsorbate surface tension effects and using the mean of adsorption/desorption part of the isotherms.

**Figure 6:**
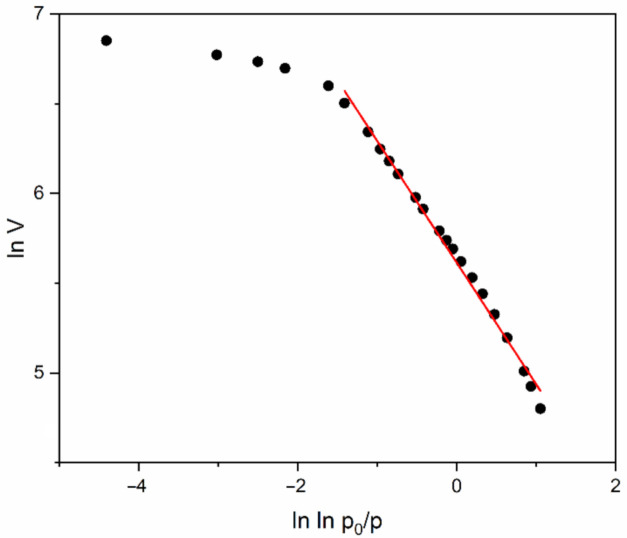
Linear fit (red line) of the low pressure adsorption region of sample MJ4 with α = 0.7.

Calculations accounted for lower pressures lead to about 15% lower values of that, presented in Table 2. The calculation of D_s_ is visualized in Figure 6, where complicated condensation phenomena in nanopores can be explained. A trend of dropping of the surface fractal dimension with an increase in the TMCS/TEOS molar ratio is visible. The TMCS addition leads to a decrease in S_BET_, combined with an increase in D_av_ and V_t_. Such observations can be qualitatively explained with repulsive steric interactions during pore formation.

## 3. Materials and Methods

### 3.1. Materials and Reagents

Tetraethoxysilane (TEOS) (Sigma, St. Louis, MO, USA), absolute ethanol (abs EtOH 99.6%) (Sigma), distilled water, trimethylchlorosilane (TMCS) (Sigma) were used in this study; the acetone, n-hexane, HCl, and ammonia used here were provided by a local supplier. All the reagents used were of analytical grade and were used without further purification.

### 3.2. Aerogel Synthesis

**Figure 7:**
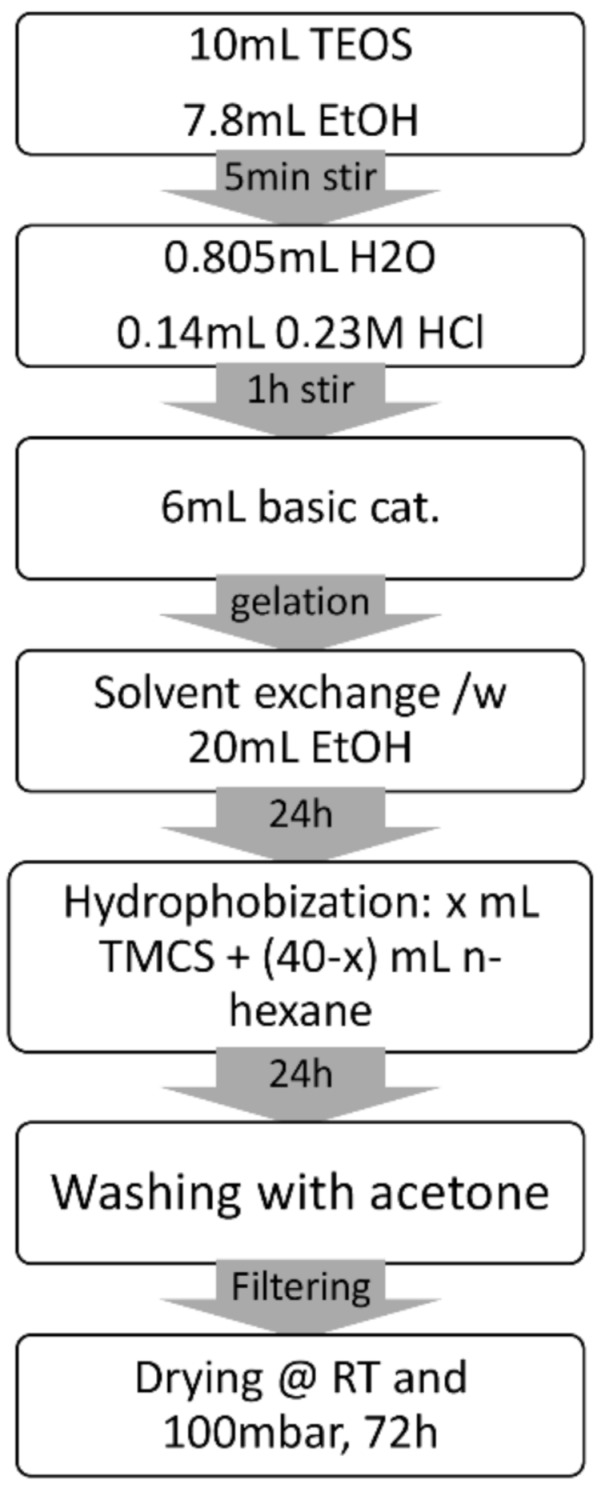
Sol-gel preparation procedure used in the present investigation. Compared to our recent published results, all steps are performed at room temperature and the solvent exchange is two times shorter.

The silica samples with different degrees of hydrophobicity (see Table 1) were synthesized via a procedure similar to that described in [20]. The scheme developed here is shown in Figure 7. TEOS 10 mL and 7.8 mL abs EtOH were mixed in a plastic PP container with a sealing cap and stirred for 5 min. Then 0.805 mL distilled water and 0.140 mL 0.23 M HCl acid catalyst were added and the mixture was stirred for 1 h at room temperature. The water:TEOS molar ratio was n_H2O_:n_TEOS_ = 1.17. After that, 6 mL ammonia based alkaline catalyst (pH 11) was added and the sol formed a gel in about 10 min. The freshly formed gel was topped with 20 mL abs EtOH and left to age for 24 h to perform solvent exchange. The ethanol was decanted and the gel was broken into a few large chunks. Surface-modifying solution was poured over the gel pieces. The solution was created by mixing x mL trimethylchlorosilane (TMCS) and 40 − x mL n-hexane. The volume of TMCS x was varied for each sample and x = 0, 2, 4, 6, 8 for samples MJ0, MJ2, MJ4, MJ6 and MJ8, respectively. The samples were left in the solution for 24 h, after which they were filtered and washed thoroughly with acetone to remove any unreacted species and waste products before the process of subcritical drying. The wet gels were placed in a vacuum oven Nüve EV 018 for 72 h. The chamber of the oven had a volume of 15,000 cm^3^. The oven was equipped with a LABOR port diaphragm pump with a delivery of 30 L/min and the power output of 300 W. The minimum achieved pressure was 100 mbar and it was maintained throughout the whole drying process.

### 3.3. ATR-IR Spectroscopy

All the attenuated total reflection infrared (ATR-IR) spectra were taken on a Bruker ALPHA II Platinum (Billerica, MA, USA)—ATR spectrophotometer equipped with a diamond crystal accessory. The spectra were taken with resolution of 1 cm^−1^ and 64 scans per sample and were normalized by the most intensive band with a maximum at 1060 cm^−1^. The spectra of the silica gels were mathematically treated via a non-linear least squares fit of the region 1300–900 cm^−1^ with overlapping five or six Gaussian functions to describe the 1260 ν_as_(Si-C-H); the longitudinal and transversal splitting of the antisymmetric ν_as_(Si-O-Si) vibration of the four- and six-membered siloxane rings LO_6_, LO_4_, TO_4_, TO_6_; and the 950 cm^−1^ ν_as_(Si-OH) band [11]. The mean R^2^ factor of the calculations was 0.999.

### 3.4. Texture Characteristics

The texture characteristics of the aerogel powders were determined by low-temperature (77.4 K) nitrogen adsorption in a Quantachrome Instruments NOVA 1200e (Boynton Beach, FL, USA) instrument. The nitrogen adsorption–desorption isotherms were analyzed to evaluate the following parameters: the specific surface areas (S_BET_) were determined on the basis of the BET equation, and the total pore volumes (V_t_) and associated average pore diameters D_av_ were estimated at a relative pressure close to 0.99. All the samples were outgassed for 16 h in vacuum at 150 °C before the measurements. The pore size distributions (PSD) were calculated by Nonlocal Density Functional Theory (NLDFT) method using equilibrium models with cylindrical pores in silica, and a hysteresis loop analysis of the isotherms was performed to obtain information about the pore shape [40]. The Frenkel–Halsey–Hill (FHH) theory describing the multilayer adsorption of a gas on a surface was used to determine the fractal surface dimension D_s_ [38,43].

## 4. Conclusions

A new approach to describe the hydrophobization of sol-gel silica with TMCS, based on the relative intensities of the ATR-IR peaks, is proposed. It is shown that the intensity of the ν_as_(Si-OH) decreases by hydrophobization, together with an increase in the intensities of the ν_s_(Si-CH) peaks. A rearrangement of the four- and six-membered siloxane rings during hydrophobization is detected following their ATR-IR spectral intensities. The dependence of degree of hydrophobicity on texture properties is demonstrated, and a reproducible laboratory room temperature technology leading to powders with a specific surface area of about 800–1000 m^2^/g and a mean pore diameter of 4–7 nm is given, which is useful for biological applications. A hysteresis loop analysis of 77 K adsorption–desorption isotherms is performed, and a pore architecture based on bottle-like pores and slit-like pores is proposed. Surface fractal dimensions D_s_ = 2.80–2.50 are calculated, depending of the degree of hydrophobicity. The hydrophobization leads to a decrease in S_BET_, combined with an increase in D_av_ and V_t_.

## Figures and Tables

**Table 1:** Sample notations, trimethyl chlorosilane (TMCS)/tetraethoxysilane (TEOS) molar ratio, and bulk density of the investigated aerogel powders.

Sample	α	ρ g/cm^3^
MJ0	0	0.45
MJ2	0.352	0.33
MJ4	0.700	0.23
MJ6	1.055	0.18
MY8	1.407	0.20

**Table 2:** Data extracted from the non-linear deconvolution of the IR spectra of the samples in the region 1300–900 cm^−1^. The relative area of the IR-bands band of samples in the region of the Si-O-Si and Si-OH vibrations.

Sample; TO/LO Splitting	Peak Index	AreaIntg, -	FWHM cm^−1^	Max Height -	Center cm^−1^	AreaIntg, %
MJ0 160.3 cm^−1^	1	9.10	60.82	0.14	948.81	9.40
	2	21.36	42.05	0.48	1040.84	22.06
	3	45.10	58.38	0.73	1070.83	46.58
	4	19.37	92.53	0.20	1131.65	20.01
	5	1.90	54.55	0.03	1201.10	1.96
	6	0.00	0.00	0.00	0.00	0.00
MJ2 163.8 cm^−1^	1	2.10	55.53	0.04	956.01	2.61
	2	31.03	44.99	0.65	1045.12	38.58
	3	35.47	52.48	0.63	1075.94	44.10
	4	10.79	94.36	0.11	1143.47	13.42
	5	0.95	42.67	0.02	1208.96	1.18
	6	0.09	6.84	0.01	1256.28	0.11
MJ4 161.8 cm^−1^	1	1.06	45.96	0.02	955.75	1.42
	2	10.25	38.17	0.25	1043.31	13.71
	3	56.78	64.25	0.83	1063.39	75.93
	4	4.72	60.74	0.07	1153.59	6.31
	5	1.81	49.63	0.03	1205.14	2.42
	6	0.16	7.89	0.02	1255.53	0.21
MJ6 165.7 cm^−1^	1	0.53	46.40	0.01	957.00	0.72
	2	14.51	38.01	0.36	1045.07	19.82
	3	50.91	59.74	0.80	1068.75	69.53
	4	5.86	72.22	0.08	1155.84	8.00
	5	1.27	44.57	0.03	1210.80	1.73
	6	0.15	7.57	0.02	1255.78	0.20
MJ8 159.1 cm^−1^	1	0.85	48.67	0.02	958.34	1.13
	2	9.93	37.15	0.25	1044.92	13.30
	3	57.41	64.44	0.84	1065.19	76.92
	4	4.24	55.74	0.07	1154.85	5.68
	5	2.04	50.21	0.04	1204.04	2.73
	6	0.18	7.89	0.02	1255.29	0.24

**Table 3:** Texture properties of the investigated samples. Specific surface area (S_BET_), total pore volume (V_t_) and associated average pore diameter (D_av_), fractal surface dimension (D_s_), and hydrophobization degree (α) are shown.

Sample	S_BET_ m^2^/g	V_t_ cm^3^/g	D_av_ Nm	D_s_	α
MJ0	1008	1.01	4.0	2.75	0
MJ2	888	0.83	4.6	2.80	0.352
MJ4	860	1.47	6.0	2.60	0.7
MJ6	872	2.06	7.0	2.55	1.055
MY8	862	2.51	7.0	2.50	1.407

## Data Availability

The data presented in this study are available on request from the corresponding author.
